# Hypoxia-mediated Krebs von den Lungen-6 expression in breast cancer: Implications for tumor invasion and metastasis

**DOI:** 10.3892/or.2025.9040

**Published:** 2025-12-19

**Authors:** Taisei Ichihara, Yoshihiro Hayashi, Takuya Sato, Mitsuko Iguchi, Makoto Toi, Hironaga Satake, Ichiro Murakami

**Affiliations:** 1 Department of Medical Oncology, Kochi Medical School Hospital, Nankoku-shi, Kochi 783-8505, Japan; 2 Department of Pathology, Kochi Medical School, Nankoku-shi, Kochi 783-8505, Japan; 3 Science Research Center, Kochi University, Nankoku-shi, Kochi 783-8505, Japan; 4 Diagnostic Pathology Department, Kochi Medical School Hospital, Nankoku-shi, Kochi 783-8505, Japan

**Keywords:** breast cancer, KL-6, hypoxia, HIF-1α, TME, invasion, metastasis

## Abstract

Krebs von den Lungen-6 (KL-6), a high-molecular-weight glycoprotein, is frequently elevated in patients with cancer; however, its precise role in the clinical progression of breast cancer (BC) remains unclear. Tumor hypoxia has been recognized as a critical driver of cancer progression. The present study aimed to investigate the effects of hypoxia on KL-6 expression and its contribution to the invasive behavior of BC cells. Immunohistochemical analysis of KL-6 and hypoxia-inducible factor-1α (HIF-1α) was performed in 30 clinical BC tissue samples, and their expression levels were correlated with patient outcomes. BC cell lines (MCF-7 and MDA-MB-231) were used for *in vitro* analyses, including immunofluorescence, western blotting, wound healing assays, and three-dimensional spheroid cultures under normoxic and hypoxic conditions, as well as chemically induced hypoxia using cobalt chloride (CoCl_2_). The subcellular localization of KL-6 was further examined through immunoelectron microscopy. In clinical specimens, high KL-6 expression was significantly associated with increased recurrence or metastasis, as was elevated HIF-1α expression. Although MCF-7 cells exhibited higher basal KL-6 expression, marked upregulation was observed in MDA-MB-231 cells under hypoxic spheroid conditions, where KL-6 co-localized with HIF-1α, particularly within invasive cellular protrusions. Functional inhibition of KL-6 suppressed the migration of MCF-7 cells. Treatment with CoCl_2_ significantly induced KL-6 and HIF-1α expression in MCF-7 cells. Ultrastructural analysis confirmed the localization of KL-6 on cell membranes and within protrusive structures. Collectively, these findings demonstrated that hypoxia enhances KL-6 expression in BC cells, partly mediated by HIF-1α, and that KL-6 contributes to tumor cell invasion.

## Introduction

Breast cancer (BC) remains the leading cause of cancer-related incidence and mortality among women worldwide and is characterized by substantial biological heterogeneity (1). This heterogeneity significantly influences therapeutic responses and clinical outcomes, posing a major challenge to the implementation of personalized medicine (2,3). The progression of BC, particularly invasion, metastasis and therapeutic resistance, results not only from genetic mutations within cancer cells but also from complex interactions with the tumor microenvironment (TME) (4,5).

In clinical oncology, tumor-associated elevations of serum Krebs von den Lungen-6 (KL-6) levels are commonly observed (6). KL-6, a high-molecular-weight mucin glycoprotein linked to mucin 1 (MUC1), is primarily used as a biomarker for interstitial pneumonia (7). Although KL-6 is considered to be co-expressed with membrane-bound MUC1 in tumor tissues, a previous study indicated its potential role in facilitating invasion and metastasis in pancreatic cancer; however, its functional significance in other malignancies remains unclear (8).

Tumor hypoxia and the subsequent activation of hypoxia-inducible factor (HIF) are key central drivers of cancer cell invasion, metastasis and TME remodeling (9–11). Notably, MUC1 expression is upregulated by HIF-1α under hypoxic conditions in models of lung adenocarcinoma and renal cell carcinoma (12–14). Although HIF-1α-mediated regulation of MUC1 has been established, the impact of hypoxia on KL-6 expression has not yet been reported.

The present study aimed to investigate the effects of hypoxia on KL-6 expression and its association with invasive behavior in BC cells and spheroid models. Hypoxia-induced changes in KL-6 expression were examined in triple-negative BC cell lines, with particular attention to membrane localization and association with invasion-associated phenotypes, using immunostaining, western blot analysis and electron microscopy. Additionally, the interplay between KL-6 expression and HIF-1α activity was investigated to elucidate the role of KL-6 in the hypoxic response. These findings provide novel insights into the regulatory mechanisms underlying KL-6 expression and its contribution to BC invasion and metastasis, suggesting its potential as a prognostic biomarker.

## Materials and methods

### Clinical data of patients with BC

The clinical data of 30 patients with early-stage BC who underwent treatment at Kochi Medical School Hospital (Nankoku, Japan) from January 2015 to December 2019 and completed at least 1 year of follow-up were retrospectively reviewed. All 30 patients were women, with a median age of 64 years (range, 40–91 years). The study was reviewed and approved by the Ethical Review Board of Kochi Medical School (approval no. 2023-146; April 19, 2024), which waived the requirement for obtaining individual written informed consent and approved the use of an opt-out procedure as an alternative. All patients underwent surgical treatment, and the resected tissues were formalin-fixed at room temperature (about 20–25°C) in 20% neutral buffered formalin for 12–24 h and then embedded in paraffin blocks. Immunohistochemical (IHC) evaluations of the estrogen receptor (ER), progesterone receptor (PR), and human epidermal growth factor receptor type 2 (HER2) status were obtained from clinicopathological reports at the time of initial diagnosis. ER and PR positivity was defined as positive staining in ≥1% of tumor cells. HER2 expression was evaluated using a four-tiered scale (0, 1+, 2+, or 3+) in accordance with the 2018 American Society of Clinical Oncology/College of American Pathologists guidelines. Patients with a score of 2+ were re-evaluated by *in situ* hybridization, and HER2 positivity was defined as a score of 3+ or a positive *in situ* hybridization result.

### KL-6 IHC evaluation

A total of 9 tissue samples obtained from patients who developed metastases or recurrence within the 1-year follow-up period were sectioned into 4-µm-thick slices and subjected to heat-induced antigen retrieval using ULTRA Cell Conditioning 1 Solution (Roche Tissue Diagnostics) at 95°C for 30 min. IHC staining was performed with an anti-KL-6 mouse monoclonal antibody (cat. no. LNM-KR-067; Cosmo Bio Co., Ltd.) using the Ventana automated system at a dilution of 1:2,000, with incubation at 4°C overnight (18 h). A total of 21 consecutive tissue samples from patients without metastasis or recurrence were processed identically and served as controls. KL-6 expression was evaluated using the Allred scoring system (13,14). Briefly, the proportion of positively stained cells was scored as follows: 0 (none), 1 (<1%), 2 (1–10%), 3 (11–33%), 4 (34–66%) and 5 (≥66%). Staining intensity in the most predominant areas was rated as 0 (none), 1 (weak), 2 (intermediate), or 3 (strong). The Allred score (range: 0–8 points) was calculated by summing the proportion and intensity scores. Immunostaining assessments were independently performed by two experienced pathologists (Mitsuko Iguchi and Makoto Toi).

### Fluorescent immunocytochemical staining

For immunocytochemical staining, MCF-7 and MDA-MB-231 cells (Health Science Research Resources Bank), each at a density of 4×10^4^ cells/ml, were cultured in slide chambers, fixed with 4% paraformaldehyde (PFA; Nacalai Tesque, Inc.) at 4°C for 30 min, and washed with phosphate-buffered saline (PBS). The cells were then incubated overnight at 4°C with an anti-KL-6 mouse monoclonal antibody diluted at 1:2,000, followed by incubation with fluorescein isothiocyanate-conjugated anti-mouse immunoglobulin (IgG) antibodies (cat. no. F-2761; 1:200; Molecular Probes; Thermo Fisher Scientific, Inc.) for 1 h at room temperature. Nuclei were counterstained with 4′,6-diamidino-2-phenylindole (DAPI; 0.001 mg/ml; MilliporeSigma). Fluorescence images were acquired using a BX53 fluorescence microscope (Olympus Corporation).

### Western blot analysis

Total protein was extracted from cells using a radioimmunoprecipitation assay buffer (FUJIFILM Wako Pure Chemical Corporation). Protein concentrations were measured using the Pierce BCA Protein Assay Kit (Thermo Fisher Scientific, Inc.). Equal amounts of protein (20 µg per lane) were separated by 10% sodium dodecyl-sulfate polyacrylamide gel electrophoresis (Bio-Rad Laboratories, Inc.) and transferred to polyvinylidene difluoride membranes using the Trans-Blot Turbo Transfer System (Bio-Rad Laboratories, Inc.). Membranes were blocked at room temperature for 1 h with Blocking One (cat. no. 03953-93; Nacalai Tesque, Inc.) and subsequently incubated overnight incubation at 4°C with an anti-KL-6 mouse monoclonal antibody (1:2,000) and a glyceraldehyde 3-phosphate dehydrogenase mouse monoclonal antibody (1:2,000; cat. no. 20035, ProMab Biotechnologies, Inc.). Subsequently, membranes were incubated for 1 h at room temperature with a horseradish peroxidase (HRP)-conjugated anti-mouse polyclonal antibody (1:2,000; cat. no. P0447; Dako, Agilent Technologies, Inc.). Protein bands were visualized using Enhanced Chemiluminescence Prime Western Blotting Detection Reagents (Amersham; Cytiva) and detected with an LAS-4000 Lumino-Image Analyzer (FUJIFILM Wako Pure Chemicals Corporation).

### Wound healing assay

Wound healing assays were performed as previously described (15). Trypsinized MCF-7 cells were counted using a C-Chip™ disposable hemocytometer (SKC, Inc.; Thermo Fisher Scientific, Inc.), seeded at a density of 2×10^4^ cells/ml into 35-mm diameter dishes, and incubated at 37°C for 48 h. Upon reaching ~90% confluency, three linear scratches were created in the cell monolayer of each dish using a 20-µl pipette tip. Phase-contrast images were captured at 0, 12, 24 and 36 h post-scratch at 50 randomly marked locations per 35-mm dish using an Olympus CKX41 microscope (original magnification, ×200; Olympus Corporation). The scratch areas devoid of cell migration were quantified using the ImageJ software version 1.53 (National Institutes of Health). The average migration across the 50 sites per dish was calculated. The relative cell migration rates at each time point were normalized to the 0-h measurement, which was set as 1.0.

### Hematoxylin-eosin and IHC staining of MDA-MB-231 cells cultured in Cell Matrix^®^

MDA-MB-231 cells (1×10^4^ cells/ml) were prepared as described in *Fluorescent immunocytochemical staining*. Cellmatrix (Nitta Gelatin Inc.) was prepared according to the manufacturer's instructions. Each cell group was mixed with 500 µl of Cellmatrix^®^ and plated into 35-mm dishes pre-coated with Cellmatrix, followed by incubation at 37°C for 30 min. The gels were overlaid with 200 µl of Dulbecco's Modified Eagle Medium (DMEM; MilliporeSigma) and cultured at 37°C for 10 days. After incubation, the gels were fixed overnight in 20% buffered formaldehyde at room temperature and embedded in paraffin. Hematoxylin-eosin staining was performed using Mayer's hematoxylin (10 min) and 1% eosin (5 min) at room temperature. IHC staining for KL-6 and HIF-1α was subsequently performed. Paraffin-embedded tissue sections (4-µm thick) were subjected to antigen retrieval by immersion in Immunosaver (Nisshin EM Co., Ltd.) at 98°C for 30 min. Endogenous peroxidase activity was quenched by incubation in 0.3% hydrogen peroxide in methanol for 10 min at room temperature. Then, sections were blocked with Blocking One for 1 h at room temperature and incubated overnight at 4°C with the following antibodies: anti-KL-6 mouse monoclonal antibody (1:2,000) and anti-HIF-1α mouse monoclonal antibody (1:100; cat. no. AP2054a, Abgent, Inc.). After washing with PBS, the sections were incubated for 1 h at room temperature with Universal ImmPRESS HRP Reagent (MP-7500; Vector Laboratories, Inc.), followed by additional PBS washes. Color development was achieved using the DAB Peroxidase Substrate Kit (SK-4100; Vector Laboratories, Inc.), and the nuclei were counterstained with Mayer's hematoxylin for 1 min at room temperature. Optical microscopic images were captured using an Olympus BX53 microscope equipped with the cellSens imaging system (Olympus Corporation).

### Spheroids of MCF-7 and MDA-MB-231 cells

A total of 1×10^4^ parental, MCF-7 and MDA-MB-231 cells per well were counted as aforementioned and seeded into PrimeSurface^®^ 96-well ultra-low attachment round-bottom plates (Sumitomo Bakelite Co., Ltd.). The cells were cultured at 37°C in a 5% CO_2_ atmosphere for 6 days to form multicellular spheroids. Following culture, spheroids were fixed and subjected to IHC staining for KL-6 and HIF-1α, as aforementioned and observed under an Olympus BX53 microscope.

### Hypoxia induction experiment using MDA-MB-231 cells

Hypoxia-induced changes in KL-6 protein expression were evaluated in MDA-MB-231 cells using immunocytochemistry. Cells (1×10^4^) were cultured for 2 days in three 2-well slide chambers (cat. no. 192-002; Watson Co., Ltd.). In each chamber, one well was treated with cobalt chloride (CoCl_2_) to induce hypoxia, whereas the other well served as an untreated control. The cells were incubated in DMEM supplemented with CoCl_2_ (2 µmol/l; cat. no. 15862-1ML-F; MilliporeSigma) at 37°C for 8 h. Following incubation, the cells were washed twice with PBS and fixed in 4% PFA at 4°C for 30 min. Each of the three slides was then assigned to a specific assay: One for KL-6 immunocytochemical staining, one for HIF-1α immunocytochemical staining, and one for hypoxia detection. Hypoxia was visualized using the LOX-1 hypoxia probe (cat. no. NC-LOX-1S; MBL Co., Ltd.). Briefly, after the 8-h incubation, LOX-1 was added to the DMEM at a concentration of 10 µl/ml. The cells were subsequently fixed again in 4% PFA (4°C for 30 min), washed twice with PBS, counterstained with DAPI, and examined under a fluorescence microscope.

### Immunoelectron microscopy

Immunoelectron microscopy was performed as previously described (16). MDA-MB-231 cells and spheroids were cultured on chamber slides or PrimeSurface 96-well ultra-low attachment plates and fixed with 4% PFA containing 0.01% glutaraldehyde (Wako Pure Chemical Industries) at room temperature for 10 min. Endogenous peroxidase activity was blocked by incubating the samples in methanol containing 0.3% H_2_O_2_ for 10 min. After washing with PBS, non-specific binding was blocked by incubation in PBS supplemented with 10% normal goat serum for 10 min. The samples were then incubated overnight at 4°C with a mouse monoclonal antibody against KL-6. After washing with PBS, the slides were incubated at room temperature for 60 min with the ImmPRESS detection reagent (Vector Laboratories, Inc.). The peroxidase reaction was visualized using DAB substrate solution (Vector Laboratories, Inc.) for 10 min. After several rinses with distilled water, the slides were treated with 0.01% aqueous hydrogen tetrachloroaurate(III) tetrahydrate (HAuCl_4_·3H_2_O; MilliporeSigma) for 10 min, rinsed again in distilled water, and dried using an air blower. The slides were then incubated in a humid chamber at 37°C for 15 h, followed by air-drying. As a control, parallel slides were incubated under dry air conditions. All samples were examined under a JSM-6010LV microscope (JEOL, Ltd.).

### Statistical analyses

All quantitative data were obtained from at least three independent experiments and are expressed as the mean ± standard deviation. To compare KL-6-positive cell rates between the MCF-7 and MDA-MB-231 cell lines, Levene's test was initially used to assess the homogeneity of variances. As unequal variances were observed, Welch's t-test was applied for statistical comparison. Western blotting was performed to evaluate the KL-6 expression in MCF-7 cells treated with or without CoCl_2_, a chemical hypoxia-inducing agent. The Shapiro-Wilk test confirmed normal distribution in both groups, whereas Levene's test revealed unequal variances; therefore, Welch's t-test was used for statistical comparison.

In the wound healing assay, the relative area covered by cells was measured at 0 and 36 h post-treatment. As the data from both treatment groups (KL-6 neutralizing antibody and non-immune IgG control) were not normally distributed, according to the Shapiro-Wilk test, comparisons were performed using the non-parametric Mann-Whitney U test. All statistical analyses were performed using the R software (version 4.4.3, http://www.R-project.org/) with a significance level set at α=0.05. P<0.05 was considered to indicate a statistically significant difference.

## Results

### Association between KL-6 and HIF-1α expressions and BC recurrence and metastasis

The association between BC recurrence or metastasis and the IHC expression of KL-6 and HIF-1α in surgical pathology specimens was investigated. Among the 30 patients, eight experienced recurrence or metastasis, whereas 20 showed no evidence of disease progression (Table SI).

Expression levels were quantified using the Allred scoring system (range, 0–8). Based on the observed score distribution, the top three score categories were defined as ‘high’ expression, and the remaining categories were classified as ‘low’ expression.

**Figure 1.**
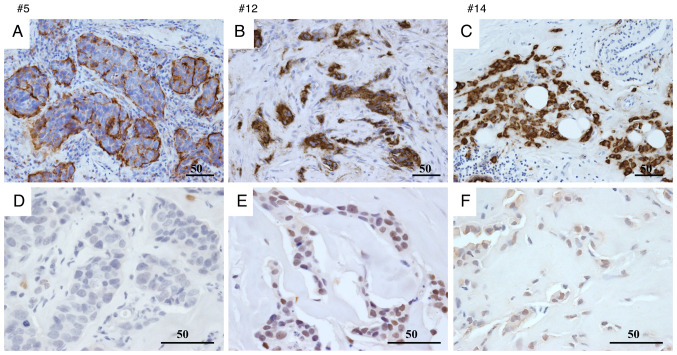
IHC expression of KL-6 and HIF-1α in breast cancer tissues. (A-F) Representative IHC images showing KL-6 (A-C) and HIF-1α (D-F) expression in tissue samples from patients 5, 12 and 14. Scale bar, 50 µm. IHC, immunohistochemical; KL-6, Krebs von de Lungen; HIF-1α, hypoxia-inducible factor-1α.

For KL-6, 7/9 patients in the recurrence/metastasis group were classified as having high expression, whereas 2/9 were classified as having low expression. This pattern is suggestive but should be interpreted with caution given the small sample (Fig. 1A-C).

For HIF-1α, 4/9 patients in the recurrence/metastasis group were classified as having high expression, whereas 5/9 were classified as having low expression. In the non-recurrence/metastasis group, 1/21 patients were classified as having high expression, whereas 20/21 were classified as having low expression. These counts indicate a numerical enrichment of higher HIF-1α expression in patients with recurrence or metastasis (Fig. 1D-F).

A single-center analysis was conducted with a limited number of events (n=9), yielding an events-per-variable (EPV) below commonly accepted thresholds for stable multivariable modeling. To prevent overfitting and unstable estimates, statistical analyses, including multivariable modeling and hypothesis testing, were not performed; accordingly, inferential statistics (for example, P-values or confidence intervals) were not reported in this subsection. The findings are therefore descriptive and hypothesis-generating and require validation in larger, multicenter cohorts with adequate EPV.

### KL-6 expression in BC cell lines

**Figure 2.**
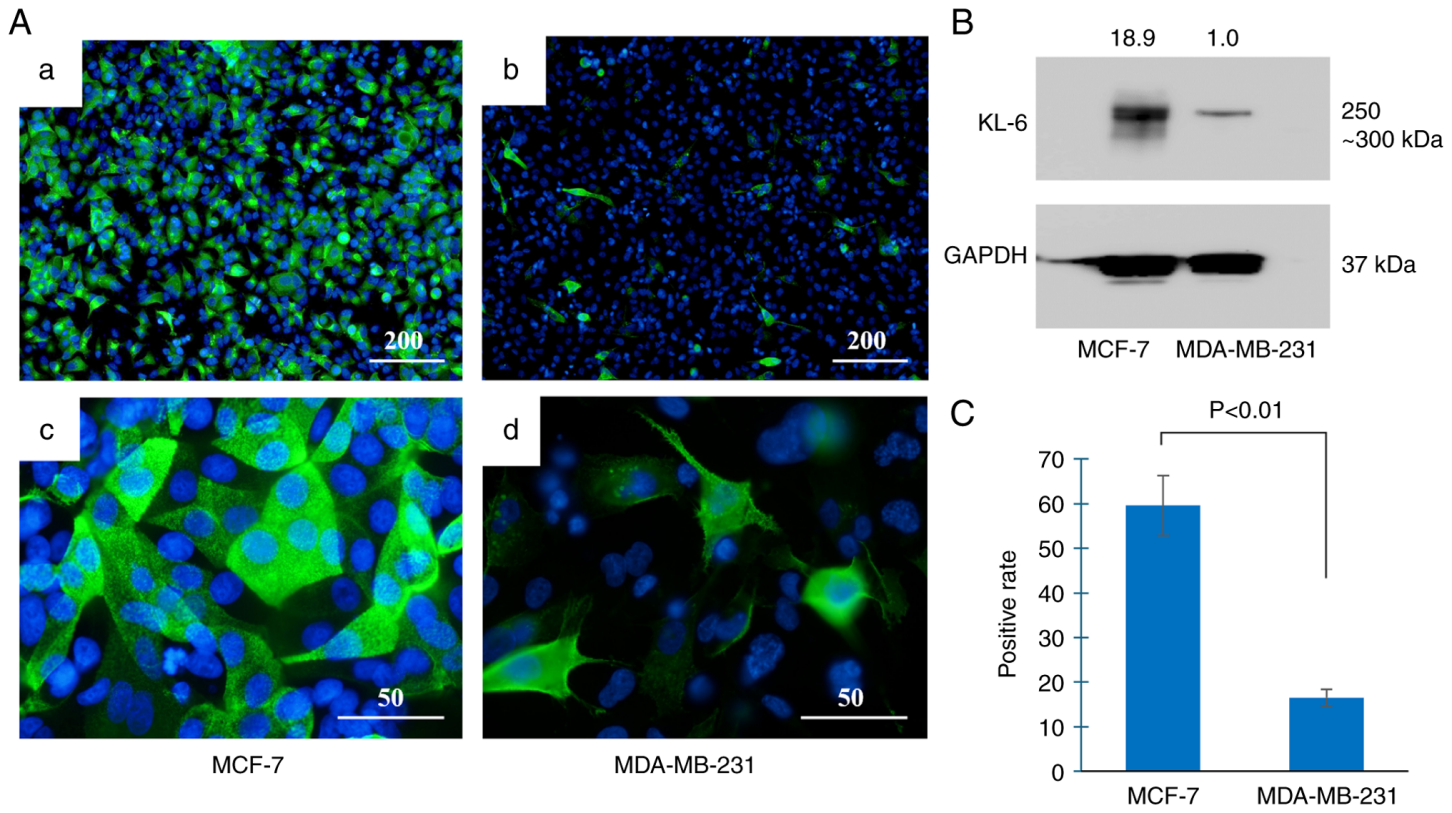
KL-6 expression in breast cancer cell lines. (A) Immunofluorescence analysis shows that most MCF-7 cells express KL-6 (a). Only a few MDA-MB-231 cells express KL-6 (b). KL-6 localization is observed as dot-like signals on the plasma membrane (c). KL-6 is localized at the membrane edges and cell protrusions (d). Scale bar, 200 µm (a and b); 50 µm (c and d). (B) Western blot analysis demonstrates lower KL-6 protein expression in MDA-MB-231 cells compared with MCF-7 cells. (C) Quantification of KL-6 positive cells in each cell line. KL-6, Krebs von de Lungen.

KL-6 protein expression in the BC cell lines was evaluated using immunofluorescence staining and western blot analysis (Fig. 2). Immunofluorescence staining (Fig. 2A) demonstrated that the majority of MCF-7 cells exhibited KL-6-positive signals (Fig. 2Aa), whereas only a small proportion of MDA-MB-231 cells were positive for KL-6 expression (Fig. 2Ab). Subcellular localization analysis revealed that KL-6 in MCF-7 cells was present as punctate staining along the cell membrane (Fig. 2Ac). By contrast, KL-6 localization in MDA-MB-231 cells was primarily observed at the membrane edges and cellular protrusions (Fig. 2Ad).

Western blot analysis (Fig. 2B) demonstrated markedly lower KL-6 expression in MDA-MB-231 cells compared with MCF-7 cells. Quantitative analysis (Fig. 2C) confirmed a higher proportion of KL-6-positive cells in MCF-7 than in MDA-MB-231 cells.

Prior to group comparison, data distribution was assessed using the Shapiro-Wilk test, which confirmed normality in both cell lines. Levene's test indicated unequal variances; therefore, Welch's t-test was applied. A significantly higher percentage of KL-6-positive cells was observed in MCF-7 cells compared with MDA-MB-231 cells (P<0.01).

### Changes in cell migration capacity following KL-6 functional inhibition: a wound healing assay

To elucidate the role of KL-6 in cell migration, a wound healing assay was conducted. MCF-7 cells were selected for this analysis due to their markedly higher KL-6 expression compared with MDA-MB-231 cells, as demonstrated using western blot analysis.

**Figure 3.**
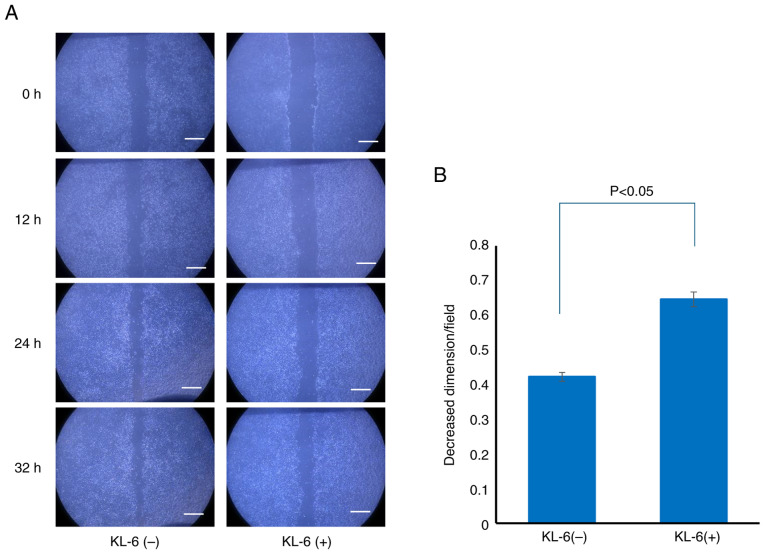
Effect of anti-KL-6 treatment on MCF-7 cell migration (wound healing assay). (A) Representative images of wound healing assays showing the impact of anti-KL-6 antibody treatment on MCF-7 cell migration. Scale bar, 500 µm. (B) Quantitative comparison of relative wound closure areas between control and anti-KL-6-treated groups. KL-6, Krebs von de Lungen.

Following the creation of a linear wound in the cell monolayer, two treatment groups were established: cells treated with a KL-6 neutralizing antibody and control cells treated with non-immune IgG. Wound closure was assessed 36 h after treatment. Substantial wound closure was observed in the control group, whereas closure was notably inhibited in the KL-6 antibody-treated group (Fig. 3A).

For quantitative analysis, the relative wound area covered at 0 and 36 h post-treatment was calculated and compared between the groups. The Shapiro-Wilk test indicated that data from both groups were not normally distributed; therefore, the non-parametric Mann-Whitney U test was employed. The analysis demonstrated that the KL-6 neutralizing antibody-treated group exhibited a significantly smaller relative covered area compared with the control group, indicating reduced migratory capacity (P<0.05; Fig. 3B).

These findings suggested that KL-6 positively regulates MCF-7 cell migration, supporting its role as a functional contributor to BC cell invasiveness.

### Expression of KL-6 and HIF-1α in the spheroid model

**Figure 4.**
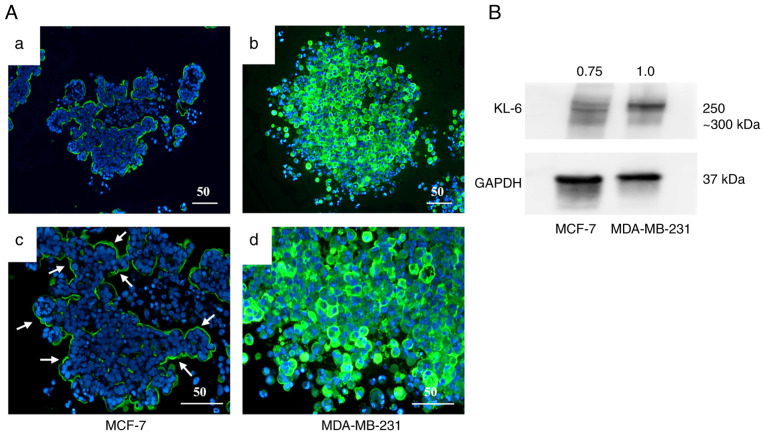
KL-6 expression in breast cancer spheroids. (A) Immunohistochemical analysis of KL-6 expression in three-dimensional spheroids. In MCF-7 spheroids, KL-6 is expressed at the spheroid periphery (a and c), with strong expression observed peripherally and internally in some spheroids (b and d). KL-6-positive regions are indicated by arrows. Scale bar, 50 µm. (B) Western blot analysis revealing higher KL-6 expression in MDA-MB-231 spheroids compared with MCF-7 spheroids. KL-6, Krebs von de Lungen.

A three-dimensional spheroid culture model was used to evaluate KL-6 expression in the BC cell lines MCF-7 and MDA-MB-231 through IHC and western blot analysis (Fig. 4).

IHC analysis (Fig. 4A) demonstrated that KL-6 positivity in MCF-7 spheroids was predominantly localized to the peripheral regions of the spheroid structure (Fig. 4Aa and c). By contrast, strong KL-6 positivity in MDA-MB-231 spheroids was observed throughout the entire spheroid, including the central regions (Fig. 4Ab and d).

Quantitative assessment by western blotting (Fig. 4B) demonstrated higher KL-6 protein levels (Fig. 4B) in MDA-MB-231 spheroids compared with MCF-7 spheroids. These findings indicated that KL-6 expression was markedly upregulated in MDA-MB-231 cells under three-dimensional culture conditions.

### Temporal changes in hypoxia induction and KL-6 expression in spheroid cultures and their involvement in cell invasion

**Figure 5.**
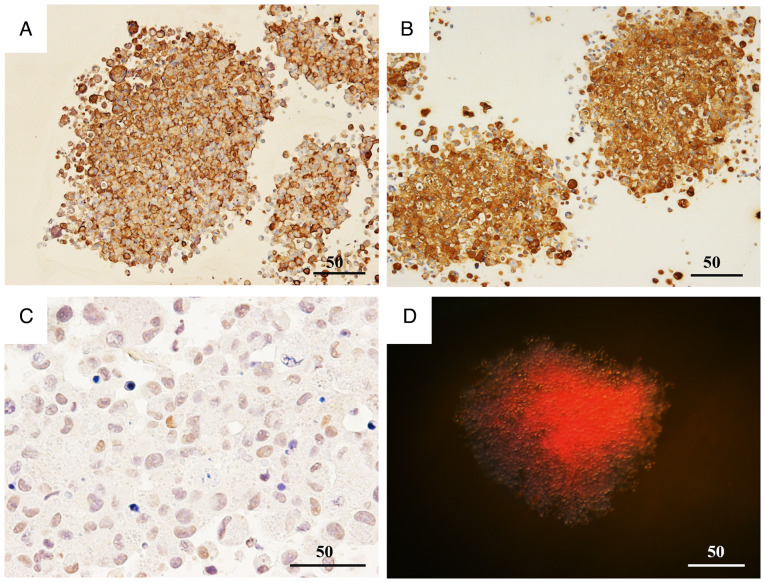
Time-dependent KL-6 expression and hypoxia in MDA-MB-231 spheroids. (A and B) IHC analysis of KL-6 expression in MDA-MB-231 spheroids at 2 days (A) and 6 days (B) after seeding, showing a mosaic-like expression pattern at both time points. (C and D) IHC staining for hypoxia-inducible factor-1α (C), a hypoxia marker, demonstrates nuclear localization at day 6 (D). Fluorescent hypoxia probe imaging confirms pronounced hypoxia within the spheroid core at day 6. Scale bar, 50 µm. KL-6, Krebs von de Lungen; IHC, immunohistochemical.

To further examine the relationship between KL-6 expression and hypoxia, the MDA-MB-231 spheroids were analyzed at various time points (Fig. 5). IHC analysis demonstrated a mosaic-like pattern of KL-6 expression 2 days after seeding (Fig. 5A); by day 6, the majority of cells exhibited KL-6 positivity (Fig. 5B). Nuclear expression of HIF-1α, a marker of hypoxia, was detected on day 6 (Fig. 5C). Furthermore, hypoxia probe-based detection demonstrated strong fluorescence signals within the spheroid core at this time point (Fig. 5D). These findings suggested that hypoxia develops progressively during spheroid culture and is associated with increased KL-6 expression in MDA-MB-231 cells.

**Figure 6.**
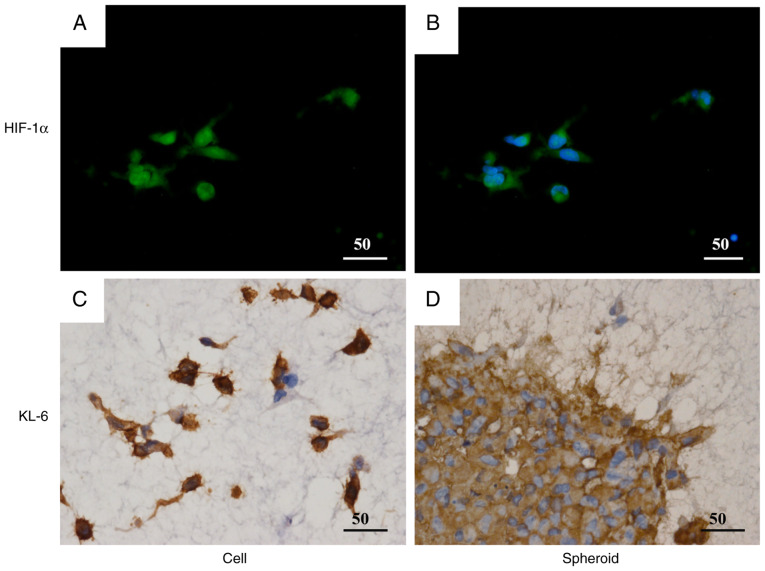
KL-6 expression in MDA-MB-231 cells and spheroids embedded in the matrix. (A and B) Immunofluorescent staining shows nuclear hypoxia-inducible factor-1α localization in most MDA-MB-231 cells within the matrix. Nuclei were counterstained with 4′,6-diamidino-2-phenylindole. (C and D) KL-6 immunohistochemical staining of MDA-MB-231 cells and spheroids embedded in the matrix. KL-6 expression is observed on cell protrusions extending from the spheroids into the surrounding matrix. Scale bar, 50 µm. KL-6, Krebs von de Lungen.

KL-6 expression was subsequently assessed in MDA-MB-231 cells and spheroids embedded within a three-dimensional extracellular matrix (Fig. 6). Immunofluorescence analysis demonstrated that most embedded cells exhibited nuclear localization of HIF-1α, as evidenced by co-localization with DAPI staining (Fig. 6A and B). IHC analysis further revealed prominent KL-6 expression in cellular protrusions extending from the spheroids into the surrounding matrix (Fig. 6C and D). These results suggested that KL-6 contributes to the invasive behavior of MDA-MB-231 cells under hypoxic conditions within a three-dimensional matrix environment.

### Induction of KL-6, HIF-1α and LOX-1 by CoCl_2_ treatment

**Figure 7.**
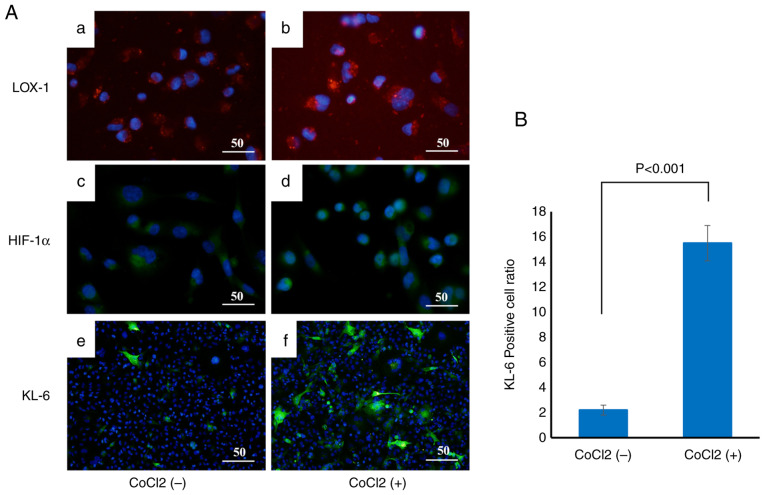
Hypoxia-induced upregulation of KL-6 expression. (A) Changes in hypoxia markers and KL-6 expression in CoCl_2_-free (a, c and e) and CoCl_2_-added (b, d and f) groups; after 12 h of CoCl_2_ addition, the fluorescence intensities of LOX-1 (a and b) and HIF-1α (c and d) increased, indicating hypoxia. Similarly, KL-6-positive cells were markedly increased in treated samples (e and f). Scale bar, 50 µm. (B) Quantification of KL-6-positive cells shows a significant increase in the CoCl_2_-treated group compared with the untreated control group. KL-6, Krebs von de Lungen; CoCl_2_, cobalt chloride.

To examine the effect of hypoxic conditions on KL-6 expression in BC cells, CoCl_2_, a chemical hypoxia inducer, was used to treat the cells, and changes in the expression of the hypoxia markers LOX-1 and HIF-1α, as well as KL-6, were evaluated by immunofluorescence staining (Fig. 7A).

MCF-7 cells were incubated with CoCl_2_ at concentrations of 2 and 4 µmol/l for 6 h at 37°C. Immunofluorescence analysis demonstrated that the fluorescence intensities of LOX-1 (Fig. 7Aa and b) and HIF-1α (Fig. 7Ac and d) were markedly increased in the CoCl_2_-treated groups, indicating effective induction of hypoxic conditions. KL-6 fluorescence was also significantly enhanced in CoCl_2_-treated cells (Fig. 7Af) compared with the untreated controls (Fig. 7Ae).

Quantitative analysis of the proportion of KL-6-positive cells relative to the total cell population revealed a significant increase in the CoCl_2_-treated group compared with the untreated group (P<0.001), as determined by Welch's t-test (Fig. 7B).

### Morphological observation of KL-6 localization via immunoelectron microscopy

**Figure 8.**
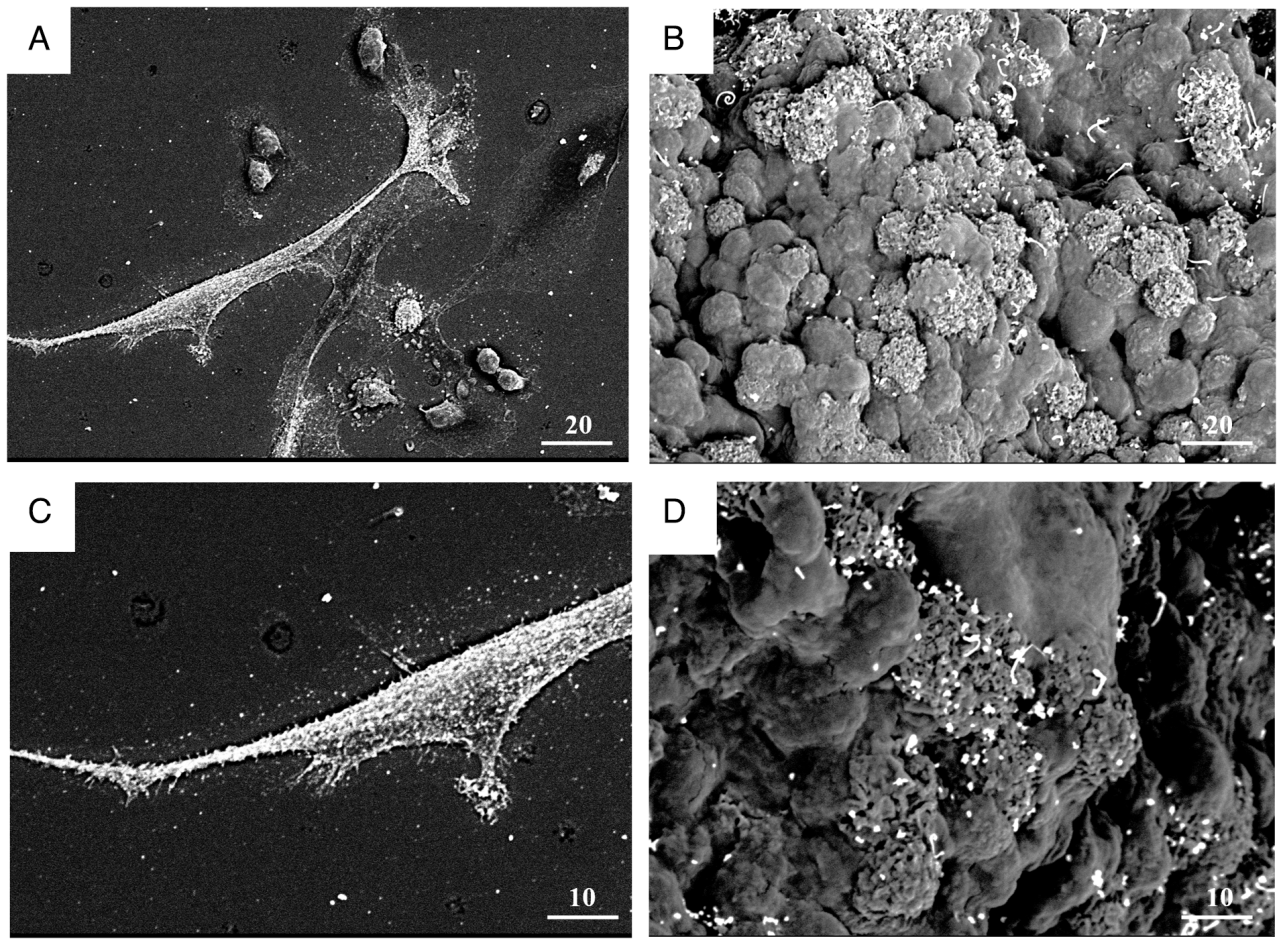
Immunoelectron microscopy of KL-6 in MDA-MB-231 cells and spheroids. (A-D) KL-6 immunogold labeling of cells (A and C) and spheroid surfaces (B and D). Positive gold particles were observed on the cell membrane, in cell protrusions, and around the cells (C). Positive staining of spheroid and cell surfaces with cell protrusions (D). Scale bar, 20 µm (A and B) and 10 µm (C and D). KL-6, Krebs von de Lungen.

To elucidate the ultrastructural localization of KL-6, immunogold labeling combined with low-vacuum scanning electron microscopy was performed on MDA-MB-231 cells and spheroids (Fig. 8).

Immunoelectron micrographs of MDA-MB-231 monolayer cells and spheroids (Fig. 8A and C) demonstrated KL-6-positive gold particles prominently localized on the cell membrane, within cellular protrusions, and at sites of cell-cell contact. Surface imaging of spheroids (Fig. 8B and D) further demonstrated the accumulation of KL-6-associated gold particles in regions enriched with protrusive structures.

## Discussion

The present study demonstrated that hypoxia increases KL-6/MUC1 expression in BC, at least in part via HIF-1α, and that KL-6 contributes to invasive behavior. Supporting evidence includes KL-6 enrichment on invasive protrusions, its upregulation in hypoxic spheroids and CoCl_2_-treated models, and the reduction of cell migration following KL-6 blockade.

In the clinical cohort, the KL-6 and HIF-1α expression levels were numerically higher in patients who experienced recurrence or metastasis, suggesting a potential association. However, this analysis was conducted in a single-center cohort with a limited number of events (n=9), resulting in an EPV below commonly accepted thresholds for stable multivariable modeling. To avoid overfitting and unstable estimates, multivariable modeling and formal hypothesis testing were not performed, and only descriptive associations were reported. Consequently, these findings are hypothesis-generating and should be interpreted with caution, as potential confounding factors such as tumor stage, treatment, and subtype could not be adjusted for in this analysis.

In the experiments of the present study (Fig. 2), the proportion of KL-6-positive cells was significantly higher in MCF-7 than in MDA-MB-231 cells (P<0.01), indicating that basal KL-6 expression is enriched in MCF-7 under the assay conditions employed. Consistent with established biology, MCF-7 cells display a luminal A epithelial phenotype characterized by strong E-cadherin and low vimentin expression. Conversely, MDA-MB-231 cells exhibit a mesenchymal program, including loss of E-cadherin, vimentin and N-cadherin expression; activation of the Ras-related C3 botulinum toxin substrate-extracellular signal-regulated kinase-nuclear factor kappa-light-chain-enhancer of activated B cells signaling pathway; and elevated matrix metalloproteinase activity. These traits support extracellular matrix degradation, stromal adhesion and motility, thereby conferring greater invasive potential (17–21). Collectively, these observations indicate that higher KL-6 expression in MCF-7 cells does not, by itself, determine invasive capacity; rather, invasion likely reflects the integrated effects of KL-6-dependent and KL-6-independent mechanisms, including epithelial-mesenchymal transition (EMT) status, NF-κB/Rac signaling, protease activity, and tumor-stroma interactions.

The structural characteristics of KL-6, particularly its sialic acid modifications, have been implicated in promoting cancer cell invasiveness and metastatic potential (22). Sialylation represents a key molecular alteration that facilitates tumor cell migration, adhesion and immune evasion (22). Consistent with other sialylated glycans, KL-6 was observed at the cell membrane in the current electron microscopy analyses (23). This localization suggests a role in mediating cell-cell adhesion and interactions with the extracellular matrix. Given the established involvement of sialylated glycans in promoting cancer cell invasiveness and dissemination, KL-6 may similarly enhance invasion and metastasis by modulating cell-cell and cell-matrix interactions within the TME (22). These findings suggest that KL-6 may function beyond its utility as a biomarker and actively contribute to malignant BC phenotypes through mechanisms characteristic of sialylated glycoconjugates (24).

IHC and western blot analyses demonstrated elevated KL-6 expression in MDA-MB-231 cell spheroids compared with conventional monolayer cultures. This observation led to the hypothesis that the increased KL-6 expression observed in the spheroid models may be attributable to the hypoxic conditions characteristic of three-dimensional cultures (25). To test this hypothesis, a chemical hypoxia model was established using CoCl_2_. Following CoCl_2_ treatment, the upregulation of HIF-1α expression was observed, confirming the successful induction of a hypoxic environment. Concurrently, a significant increase in the proportion of KL-6-positive cells was detected, indicating that KL-6 expression is inducible under hypoxic conditions. Hypoxia modulates the expression of adhesion molecules and glycosylation-related proteins, enhancing the invasive and metastatic potential of cancer cells (26,27). HIF-1α, a central regulator of the cellular hypoxic response, governs the expression of numerous tumor-associated genes (10). The present findings suggest that KL-6 may act downstream of the HIF-1α signaling, contributing to the malignant phenotype of BC cells. Additionally, sialic acid modifications, which are promoted under hypoxic conditions, have been shown to facilitate tumor invasion and metastasis; KL-6 may similarly participate in these sialylation-mediated mechanisms (22).

KL-6 is a glycan-dependent epitope on MUC1 (cluster 9). The minimal epitope recognized by the KL-6 antibody is located within the MUC1 tandem repeat bearing an α2,3-sialyl-T structure (7). Consequently, increased MUC1 expression and sialylation are expected to enhance KL-6 detection in tumor cells. Under chronic hypoxia, HIF-1α and NF-κB p65 cooperatively induce MUC1/MUC1-C, and direct genetic perturbation abolishes this response. Using a hypoxia fate-mapping/CRISPR system, knockout of HIF-1α or NF-κB p65 prevented hypoxia-induced MUC1-C accumulation (14). Functionally, either deletion of MUC1 under hypoxic conditions or pharmacologic inhibition of MUC1-C with the cell-penetrant inhibitor GO-203 increased mitochondrial reactive oxygen species in circulating tumor cells and reduced the contribution of hypoxia-experienced cells to lung metastasis *in vivo* (14).

Multiple independent lines of evidence indicate that MUC1 is a direct HIF-1 target gene (12,13,28). In clear-cell renal cell carcinoma models, stabilization of HIF-1α by CoCl_2_ led to increased MUC1 mRNA levels, whereas HIF-1α knockdown via small interfering RNA and the HIF-1 inhibitor YC-1 reduced hypoxia-induced MUC1 expression. Chromatin immunoprecipitation and electrophoretic mobility shift assay identified HIF-1α binding at two hypoxia-responsive elements within the MUC1 promoter, establishing direct transcriptional control (13). In conjunction with studies that MUC1 stabilizes HIF-1α and co-activates hypoxia-metabolic targets, these findings support the existence of a feed-forward HIF-MUC1 axis that reinforces hypoxic adaptation. Functionally, hypoxia increased invasion and migration by ~4.7-fold and 3.9-fold, respectively, whereas MUC1 knockdown significantly reversed these hypoxia-enhanced phenotypes (13). Building on these findings, a coherent model is proposed in which KL-6/MUC1 promotes invasion and metastasis through HIF-1α-dependent transcriptional control.

Sialic acid-modified mucin family molecules have been reported to alter the physical and biological properties of cell surfaces, thereby facilitating the acquisition of invasive and metastatic capabilities (29). α2,3-linked sialylation has been consistently associated with metastatic behavior across multiple tumor types. In gastric cancer, elevated α2,3-linked sialic acids correlate with increased metastasis and poorer prognosis, consistent with the present observation that the α2,3-sialylated KL-6 epitope is elevated in motile states (30). These findings support a model in which α2,3 sialylation marks an invasive phenotype. ST3GAL1 catalyzes the installation of terminal α2,3-sialic acid on O-glycans, the same linkage that defines the KL-6 (sialyl-T) motif on MUC1. Functionally, ST3GAL1 promotes migration, invasion, and TGF-β1-induced EMT in ovarian cancer (31). In melanoma, ST3GAL1 enhances dimerization and activation of the receptor tyrosine kinase AXL, increasing invasion and metastatic seeding (32). Collectively, these findings support a coherent mechanism in which a KL-6-high state reflects ST3GAL1-driven α2,3 sialylation on MUC1 and other surface proteins, thereby potentiating receptor tyrosine kinase (RTK)-centered signaling (for example, AXL) that promotes invasion (31,32). This delineates a plausible downstream pathway by which KL-6 may facilitate invasion through ST3GAL1-dependent α2,3 sialylation and subsequent activation of RTK signaling.

The present study has certain limitations. First, the relatively small sample size necessitates cautious interpretation when generalizing the observed association between KL-6 expression and recurrence or metastasis risk (29). Future studies with larger sample sizes and multi-institutional collaborations are required to validate these findings. Second, although a chemical hypoxia model (CoCl_2_ treatment) was employed, it may not fully replicate the sustained and multifactorial hypoxic conditions present within TMEs. Future studies should evaluate KL-6 expression dynamics using physical hypoxia culture systems and *in vivo* models.

Furthermore, the downstream signaling pathways and functional roles of KL-6 in cell adhesion and invasion require further elucidation. In particular, the identification of specific receptor molecules and extracellular matrix components that interact with KL-6 remains a critical challenge and is directly relevant to the development of novel therapeutic strategies for BC.

In conclusion, the present study provides novel insights into the biological significance of KL-6 in BC. Further investigations are warranted to explore its potential clinical applications.

## Supplementary Material

Supporting Data

## Data Availability

The data generated in the present study may be requested from the corresponding author.

## Abbreviations

KL-6: Krebs von den Lungen-6
MUC1: mucin 1
TME: tumor microenvironment
HIF-1α: hypoxia-inducible factor-1α
ER: estrogen receptor
PR: progesterone receptor
HER2: human epidermal growth factor receptor type2
PFA: paraformaldehyde
PBS: phosphate-buffered saline
DAPI: 4′,6-diamidino-2-phenylindole
HRP: horseradish peroxidase
CoCl_2_: cobalt chloride
EPV: events-per-variable
Rac-ERK-NF-κB: signaling pathway
EMT: epithelial-mesenchymal transition
RTK: receptor tyrosine kinase
